# *Pseudomonas* sp. ST4 produces variety of active compounds to interfere fungal sexual mating and hyphal growth

**DOI:** 10.1111/1751-7915.13289

**Published:** 2018-06-21

**Authors:** Shiyin Liu, Fei He, Nuoqiao Lin, Yumei Chen, Zhibin Liang, Lisheng Liao, Mingfa Lv, Yufan Chen, Shaohua Chen, Jianuan Zhou, Lian‐Hui Zhang

**Affiliations:** ^1^ Guangdong Province Key Laboratory of Microbial Signals and Disease Control South China Agricultural University Guangzhou China; ^2^ Integrative Microbiology Research Centre South China Agricultural University Guangzhou China

## Abstract

Sexual mating of compatible sporida is essential for *Sporisorium scitamineum* to form dikaryotic mycelia and then cause infection on sugarcane. Our previous work identified a *Pseudomonas* sp. ST4 from a soil sample, which showed a promising biocontrol potential by inhibiting the mating of *S. scitamineum* sporida and hyphal growth. In this study, we set to isolate the active compounds from *Pseudomonas* sp. ST4 through solid fermentation. High‐performance liquid chromatography (HPLC) separation coupling with bioassay showed that *Pseudomonas* sp. ST4 produced a range of antimicrobial compounds. Two of the major components were purified following acetate extraction, silica gel and HPLC separation. Nuclear magnetic resonance (NMR) and liquid chromatography–mass spectrometry (LC‐MS) analysis identified these active compounds are 4‐hydroxybenzaldehyde and indole‐3‐carbaldehyde respectively. Further analysis showed that the former compound only inhibited the hyphal growth of the fungus at a concentration of 3 mM, while the latter interfered the fungal sexual mating at a concentration of 0.6 mM and affected hyphal growth at a concentration of 2 mM. Treatment of corn plants with 3 mM indole‐3‐carbaldehyde significantly inhibited corn smut infection, with a control rate up to 94%. Further analysis of the structure and activity relationship revealed that indole has a much stronger inhibitory activity against the fungal sexual mating than indole‐3‐carbaldehyde. The results from this study provide new agents for control and prevention of the sugarcane smut disease, and the active compounds could also be used to probe the molecular mechanisms of fungal sexual mating.

## Graphical abstract

4‐hydroxybenzaldehyde and indole‐3‐carbaldehyde from Pseudomonas sp. ST4 were isolated and identified, the former of which inhibited hyphal growth of Sporisorium scitamineum at a concentration of 3 mM, and the latter of which interfered the fungal sexual mating at a concentration of 0.6 mM, and affected hyphal growth at a concentration of 2 mM and has a good control efficacy against sugarcane smut.

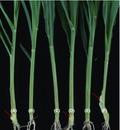

## Introduction

Sugarcane smut disease was first reported in Natal, South Africa, in 1877 (Mcmartin, 1945), and hereafter quickly spread to countries worldwide, such as Kenya, Nigeria, Mali, Tanzania, Mauritius and Rhodesia in Africa (Bock, 1964; Waller, 1967; Msechu and Keswani, 1982; Wada, 2003; Nzioki and Jamoza, 2006; Ong'Ala *et al*., 2016), Argentina, Bolivia, Brazil, Dominican, Jamaica, Louisiana, Hawaii, Colombia in America and also Australia in 1998 (Byther *et al*., 1973; Comstock and Heinz, 1977; Hoy *et al*., 1986; Victoria *et al*., 1990; Tirado‐Corbalá *et al*., 2015). In China, smut disease greatly influences sugarcane yield and could cause a huge direct economic loss up to five billion Chinese yuan every year (Shen *et al*., 2013). Currently, control of the smut disease is mainly relied on breeding of resistant sugarcane varieties, which is time‐consuming and restricted by availability of resistant parental varieties and emergence of new pathovars. In addition, a range of fungicides were also evaluated against the smut fungus (Olufolaji, 1993; Wada, 2003; Bhuiyan *et al*., 2012), but their associated environmental toxicity and quick emergency of fungicide resistance has become a public concern.

The pathogen of sugarcane smut is *Sporisorium scitamineum*, which was previously named as *Ustilago scitaminea* (Piepenbring *et al*., 2002) with a life cycle similar to those of the maize pathogens *Ustilago maydis* and *Sporisorium reilianum* (Schirawski *et al*., 2005, 2010; Kämper *et al*., 2006). As a heterothallic fungus, *S. scitamineum* produces basidiospores of two mating types. The basidiospores can grow and proliferate to generate more basidiospores through budding, which are unable to cause infections. The basidiospores could also mate with other basidiospores of different mating type to form dikaryons and then develop into hyphae, which are able to penetrate the bud scales of the sugarcane and cause smut disease (Croft and Braithwaite, 2006; Yan *et al*., 2016). Investigation of mating mechanism revealed the *b* locus as one of the important loci for fungal sexual mating process and pathogenesis (Kämper *et al*., 1995; Yan *et al*., 2016). Apparently, the fungal sexual mating is the key for the pathogenicity of *S. scitamineum,* which could be a promising target for design and developing new disease control and prevention strategies. In our previous study, we have isolated a *Pseudomonas* sp. ST4 from vegetable rhizosphere in Shantou city, Guangdong Province, China, which showed a potent inhibitory activity against the sexual mating of *S. scitamineum* without killing the fungal haploid cells (Liu *et al*., 2017). To understand the mechanism of biocontrol, we optimized the fermentation and chromatography conditions for isolation of the active compounds that inhibit the fungal sexual mating. Our results showed that *Pseudomonas* sp. ST4 produced diverse active compounds, some of which might inhibit both the sexual mating and the growth of hyphae, and some could only inhibit the sexual mating or the hyphal growth. The findings from this study provide useful clues and effective agents for the prevention and control of smut disease caused by *S. scitamineum*.

## Results

### *Pseudomonas* sp. ST4 produces diverse antimicrobial molecules

**Figure 1:**
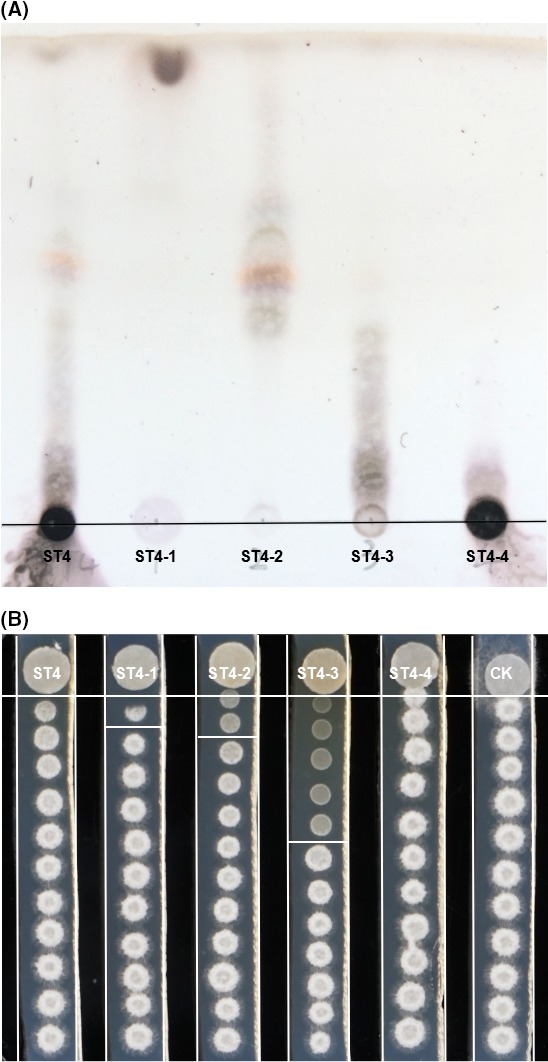
Analyses of TLC and antimating activity of fractions ST4‐1 to ST4‐4. A. After separation by silica gel column and medium pressure separator, 0.5 μl of each fraction (dissolved in MeOH in a concentration of 50 mg ml^−1^) was spotted on a aluminium plate using capillaries, which was developed by a solution of CHCl_3_/MeOH (9:1 *v/v*), and dyed by 10% C_4_H_10_O_4_S colour agent. Four fractions were obtained based on the pattern similarity in the thin layer chromatography (TLC) analysis. B. Mixture of MAT‐1 and MAT‐2 (0.5 μl of OD_600_ ≈ 1.5) was spotted on PDA slices progressively and tablet (5 mm diameter) absorbed each fraction was placed on the end of the PDA slice for antimating bioassay. The bioassay plate was incubated at 28°C for 2 days until white hypha appeared on the MeOH control (CK). Results showed that the most active fraction was ST4‐3 according to the hyphal growth inhibition distance (in horizontal white line), which was followed by fractions ST4‐2 and ST4‐1, while no inhibitory activity could be detected in fraction ST4‐4.

**Figure 2:**
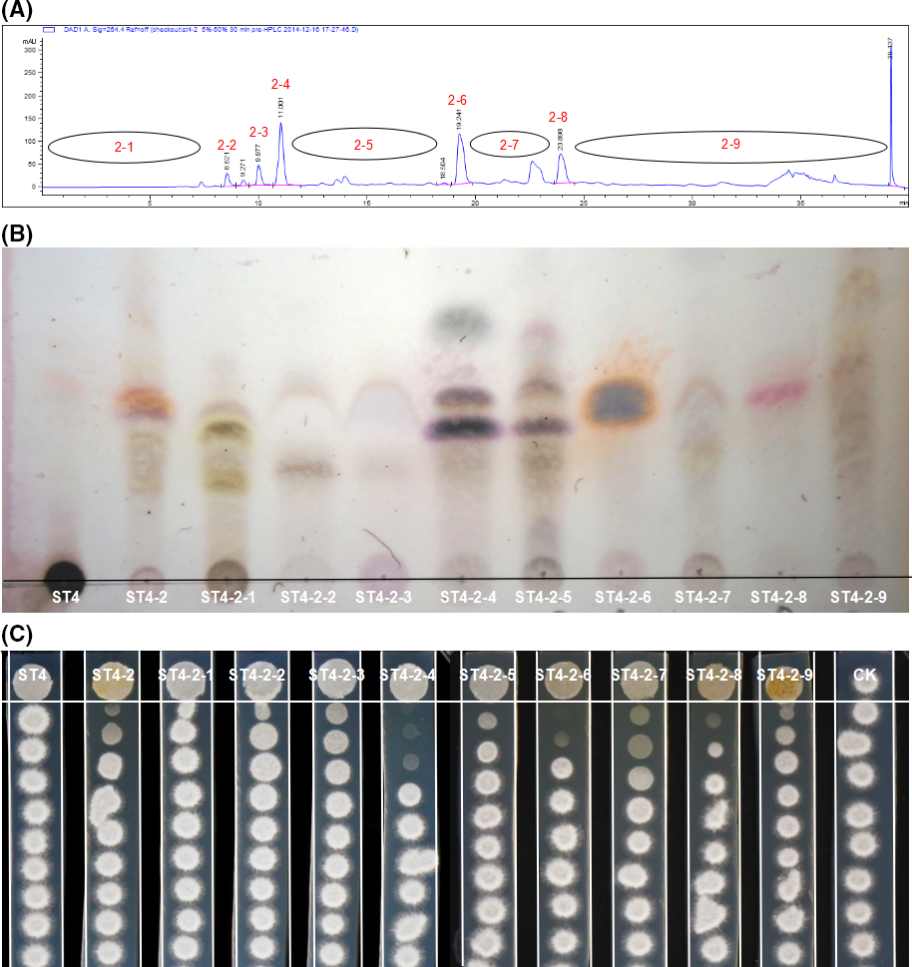
Analyses of HPLC, TLC and antimating activity of fractions ST4‐2‐1 to ST4‐2‐9. (A) The UV absorbance (λ = 254 nm) profile of the HPLC fractions of the active components after silica gel column and medium pressure separation. B. TLC analysis of each ST4‐2 fractions after HPLC separation. C. Antimating bioassay of each ST4‐2 fractions. CK, no tablet absorbed in any ST4‐2 fraction was placed on the end of the PDA slice. Bioassay results showed that fractions ST4‐2‐4 and ST4‐2‐6 had strongest inhibitory activities against the hyphal growth, fraction ST4‐2‐7 restrained the sexual mating with little inhibitory activity on the hyphal growth, and fraction ST4‐2‐8 could inhibit both sexual mating and the hyphal growth.

*Pseudomonas* sp. ST4 displayed a potent inhibitory activity on the sexual mating of *S. scitamineum,* and evidence suggests that the active components are small molecules diffusible in PDA agar medium (Liu *et al*., 2017). Thermal stability assay showed that *Pseudomonas* sp. ST4 metabolites lost the antimating activity against *S. scitamineum* haploid cell lines MAT‐1 and MAT‐2 after treatment at 100°C for 30 min (Fig. S1), and the bioactive compounds was soluble in hydrophobic ethyl acetate other than hydrophilic butanol (Fig. S2). For large scale purification, a total of 38.82 g ethyl acetate crude extract was obtained from 20 litres of *Pseudomonas* sp. ST4 spent PDA medium. The ethyl acetate crude extract were then separated in batches in a silica gel column with a Butch medium pressure filter. Four fractions were obtained based on the pattern similarity in the thin layer chromatography (TLC) analysis (Fig. 1A). The mating inhibitory activity of these four fractions were tested, and the results showed that the most active fraction was ST4‐3 according to the distance of inhibition zone, which was followed by fractions ST4‐2 and ST4‐1, while no inhibitory activity could be detected in fraction ST4‐4. Despite the fraction ST4‐3 was the most active in the mating inhibition assay, we failed to obtain any pure compound with antimicrobial activity from this fraction owing to its polarity. Considering that fraction ST4‐2 had reasonable antimating activity with moderate polarity and relatively simpler molecular pattern according to TLC assay (Fig. 1), this fraction about 2000 mg solid mixture was selected for subsequent HPLC purification. The results of semi‐preparative reversed‐phase HPLC showed that fraction ST4‐2 contained at least over two dozens of molecules according to the UV absorption peaks at OD_220_ (Fig. 2A). Based on the HPLC profile, nine fractions were collected, including ST4‐2‐1 (0–8.6 min, 74.4 mg), ST4‐2‐2 (8.6–9.4 min, 18.6 mg), ST4‐2‐3 (9.4–10.0 min, 35.7 mg), ST4‐2‐4 (10.0–11.5 min, 6.6 mg), ST4‐2‐5 (11.5–18.6 min, 37.0 mg), ST4‐2‐6 (18.6–20.0 min, 9.1 mg), ST4‐2‐7 (20.0–23.4 min, 36.8 mg), ST4‐2‐8 (23.4–24.5 min, 6.9 mg), and ST4‐2‐9 (24.5–40.0 min, 307.5 mg) (Fig. 2A). Among them, both fractions ST4‐2‐6 and ST4‐2‐8 showed only a single peak with retention times at 19.241 and 23.590 min respectively (Fig. 2A), and a pure band on the TLC plate (Fig. 2B).

Bioassay results showed that most HPLC fractions from ST4‐2 displayed antagonistic activity against the hyphal growth or sexual mating of *S. scitamineum*. Among them, Fractions ST4‐2‐4 and ST4‐2‐6 had strongest inhibitory activities against the hyphal growth with no colonies appeared near the fraction samples; fraction ST4‐2‐7 blocked the sexual mating with little inhibitory activity on the hyphal growth; whereas fraction ST4‐2‐8 inhibited both sexual mating and the hyphal growth (Fig. 2C).

### Structural characterization of the compounds in fractions ST4‐2‐6 and ST4‐2‐8

**Figure 3:**
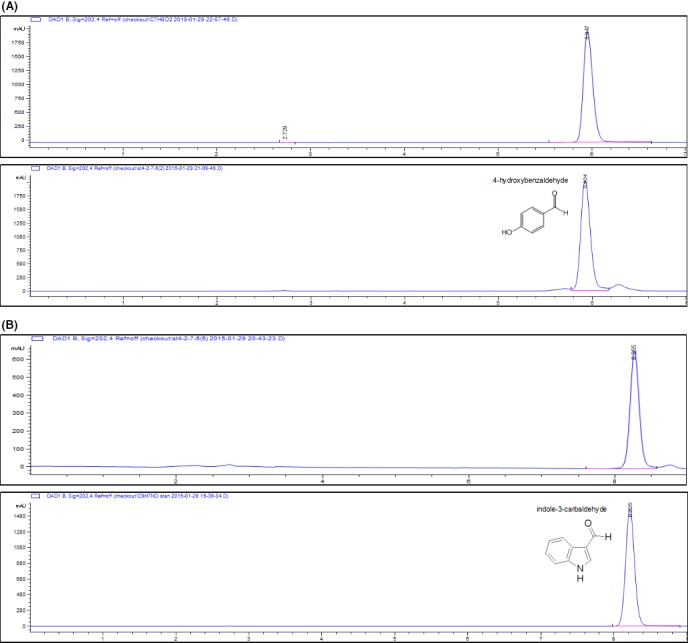
HPLC analysis of fractions ST4‐2‐6 (A) and ST4‐2‐8 (B). A. The retention time of Fr. ST4‐2‐6 is 5.9 min (top), the same as that of the standard sample of 4‐hydroxybenzaldehyde (bottom). B. The retention time of Fr. ST4‐2‐8 is 8.255 min (top), the same as that of the standard sample of indole‐3‐carboxaldehyde (bottom).

Fractions ST4‐2‐6 and ST4‐2‐8, which appeared pure in HPLC and TLC analyses, were characterized by NMR and mass spectrometry. ^1^H and ^13^C NMR spectra revealed that Fraction ST4‐2‐6 contains a benzene ring, with a hydroxyl group at α site and an aldehyde group at δ site (Fig. S4A and B). LC‐MS analysis of the compound showed a molecular weight of 122.12, identical to that of 4‐hydroxybenzaldehyde. HPLC analysis showed that the retention time of fraction ST4‐2‐6 was at 5.9 min, which was identical to that of the commercial 4‐hydroxybenzaldehyde (Fig. 3A). The above data indicate that the single peak in fraction ST4‐2‐6 is 4‐hydroxybenzaldehyde.

For fraction ST4‐2‐8, ^1^H and ^13^C NMR spectra predicted a molecular formula of C_9_H_7_NO, containing a benzopyrrole ring with an aldehyde group at γ site (Fig. S4C and D). LC‐MS analysis showed that the compound has a molecular weight of 146.15. Together, the NMR and mass spectrometry data indicate that the compound in fraction ST4‐2‐8 is indole‐3‐carbaldehyde, which was further confirmed by HPLC analysis showing the same retention time at 8.255 min of fraction ST4‐2‐8 and commercial indole‐3‐carbaldehyde (Fig. 3B).

### Indole‐3‐carbaldehyde inhibits the fungal sexual mating and fungal growth in a concentration‐dependent manner

**Figure 4:**
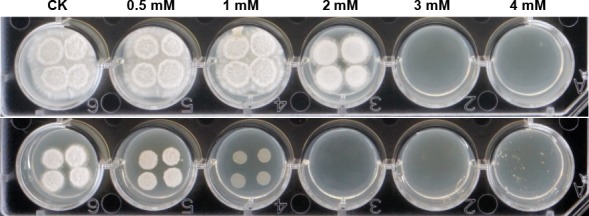
Inhibitory effects of 4‐hydroxybenzaldehyde (top) and indole‐3‐carboxaldehyde (bottom) on the sexual mating and hyphal growth of *S. scitamineum*. CK, PDA medium supplemented with MeOH. Results showed that 4‐hydroxybenzaldehyde has no antimating activity but can influence the hyphal growth at the concentration of 2 mM and above; indole‐3‐carbaldehyde can interfere the fungal mating ranging from 0.5 mM to 1 mM and inhibit the fungal growth at 2 mM.

The antimating and fungicidal activities of 4‐hydroxybenzaldehyde and indole‐3‐carbaldehyde were determined using the mixture of *S. scitamineum* haploid cell lines. The bioassay results showed that at a final concentration of < 1 mM, 4‐hydroxybenzaldehyde had no effect on the hyphal growth or sexual mating, at the concentration of 2 mM, the hyphal growth was retarded compared with that of the negative control, and at the concentration of 3 mM and above, the compound inhibited the fungal growth completely (Fig. 4A), indicating that 4‐hydroxybenzaldehyde has no antimating activity. In contrast, indole‐3‐carbaldehyde was able to interfere the fungal mating in a concentration range from 0.5 mM to 1 mM and inhibit the fungal growth at 2 mM (Fig. 4B).

### Indole‐3‐carbaldehyde provides effective control on corn smut

**Figure 5:**
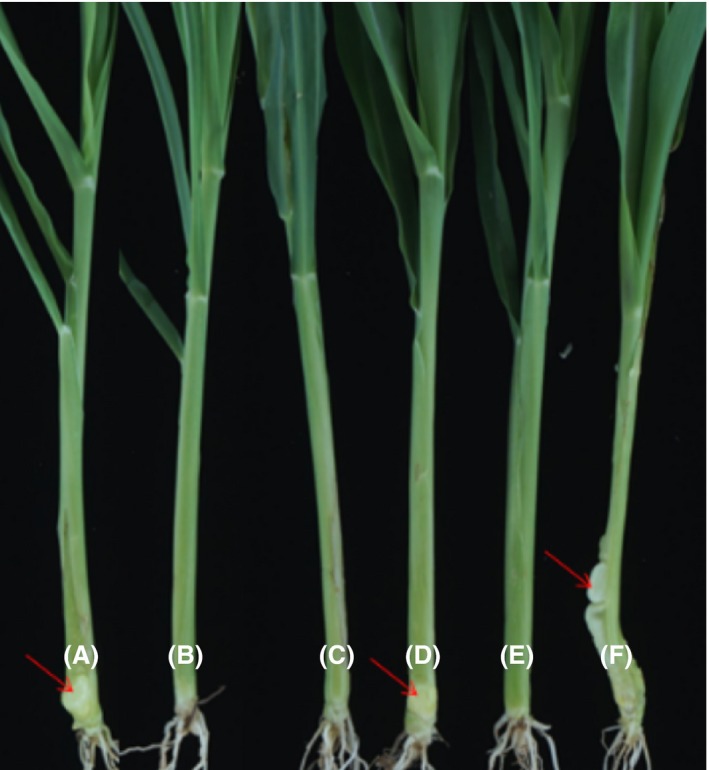
Control effects of *Pseudomonas* sp. ST4, 4‐hydroxybenzaldehyde and indole‐3‐carboxaldehyde on corn smut disease. Every 20 seedlings were injected with 500 μl of inoculums of Umn9 and Umn10 mixture containing 3 mM 4‐hydroxybenzaldehyde (A), Umn9 and Umn10 mixture containing 3 mM indole‐3‐carboxaldehyde (B), *Pseudomonas* sp. ST4 culture (OD_600_ = 0.5) containing 2% glucose (C), *Pseudomonas* sp. ST4 culture (OD_600_ = 0.5) (D), YEPS medium (E), and Umn9 and Umn10 cell mixture (F) respectively. All the seedlings were kept outdoor for 7–10 days, and disease incidence was measured. The trial was conducted twice. Results showed that 3 mM 4‐hydroxybenzaldehyde has few disease control efficiency (A), and indole‐3‐carbaldehyde at the same concentration has a good efficiency on controlling smut disease occurrence (B).

Similar to the sugarcane pathogen *S. scitamineum*,* Ustilago maydis,* which is the causal agent of the corn smut disease, also requires a sexual mating process to develop from non‐pathogenic haploid cells to pathogenic diploid filaments. Importantly, *Pseudomonas* sp. ST4 also showed a strong inhibitory effect on the mating of the haploid cell lines Umn9 and Umn10 of *U. maydis*, and 4‐hydroxybenzaldehyde and indole‐3‐carboxaldehyde displayed a similar inhibitory effect on the hyphal growth or sexual mating of *U. maydis* as against the sugarcane pathogen (Fig. S3). To test the disease control efficiency of 4‐hydroxybenzaldehyde and indole‐3‐carbaldehyde, we used the corn seedlings infected with *U. maydis* instead of the sugarcane plants infected with *S. scitamineum*, as the disease cycle of the former is greatly shorter (about 20 days) than the latter (about 7–8 months). Results showed that all the seedlings treated with *U. maydis* developed disease symptoms rapidly with typical white deformed tumours grew at the infection sites, which progressively turned into black and even burst out from leaf sheaths (Fig. 5, Table 1). Treatment of *U. maydis* infected corn seedlings using *Pseudomonas* sp. ST4 slightly reduced the disease occurrence, with a disease incidence at about 89%. However, similar to the case of sugarcane trials (Liu *et al*., 2017), when *Pseudomonas* sp. ST4 grew and inoculated with 2% glucose, its biocontrol efficiency was greatly increased with a disease incidence reduced to only about 15% (Table 1). When treated with 3 mM 4‐hydroxybenzaldehyde, all the seedlings showed disease symptoms but the tumours were smaller than the untreated control (Fig. 5). Significantly, indole‐3‐carbaldehyde has a high disease control efficiency, with < 6% of the seedlings showing small tumours at the infection sites when the compound was added at a final concentration of 3 mM (Table 1).

**Table 1:** Control efficiency of strain ST4 and its metabolites on corn smut disease

	Corn smut		
Treatment	Seedling number (diseased/all^a^)	Incidence (%)	Control efficiency^b^ (%)
YEPS medium (negative control)	0/40	0.00	–
*U. maydis* (positive control)	40/40	100	–
ST4 + *U. maydis*	32/36	88.89	11.11
ST4 + *U. maydis*+2% glucose	6/39	15.38	84.62
*U. maydis*+3 mM 4‐hydroxybenzaldehyde	40/40	100	0
*U. maydis*+3 mM indole‐3‐carboxaldehyde	2/36	5.56	94.44

a The total number of seedlings for every treatment is 40; several seedlings were naturally dead before measurement, which were not calculated for data statistics.

b Control efficiency (%) = (Incidence_positive control_‐Incidence_treatment_) ×100/Incidence_positive control_.

### Evaluation of the antimating activity of indole‐3‐carbaldehyde derivatives

Various derivatives containing indole group or carbaldehyde moiety were purchased, and their antimating activity against *S. scitamineum* were determined. Results showed that at a low concentration of 0.2 mM, indole displayed a promising activity in inhibition of the sexual mating of *S. scitamineum*, which is about threefold lower than the effective concentration of indole‐3‐carbaldehyde under the same assay conditions. Noticeably, indole did not affect sporida growth even at a high concentration up to 0.8 mM (Table 2). The minimum inhibitory concentration (MIC) of most derivatives is about 0.6 mM except indole‐4‐carbaldehyde (Table 2). For the derivatives with group substituted the carbaldehyde base at γ site, the more complicate the group, the weaker the inhibitory activity, and *vice versa*, the inhibitory activity of the reduced methanol base is stronger than that of the oxidized formic acid base. Substitution of the bases at other positions of indole group to indole 3‐carbaldehyde could increase the inhibitory effect against the hyphal growth.

**Table 2:** Inhibitory effects of indole and its derivatives on *S. scitamineum*

		0.2 mM		0.4 mM		0.6 mM		0.8 mM		MIC (mM)	
Structure	Chemical density	Mating	Growth	Mating	Growth	Mating	Growth	Mating	Growth	Mating	Growth
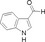	Indole‐3‐carboxaldehyde	−	−	−	−	(+)	−	(+)	−	0.6	3
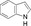 ;	Indole	(+)	−	+	−	+	−	+	−	0.2	NIA
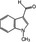	1‐methylindole‐3‐carbaldehyde	−	+	−	+	−	+	+	+	0.8	0.2
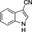	3‐cyanoindole	−	(+)	(+)	(+)	+	+	+	+	0.4	0.2
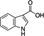	Indole‐3‐carboxylic acid	−	−	−	−	−	−	(+)	−	0.8	NIA
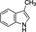	3‐methyl indole	(+)	−	+	−	+	−	+	+	0.2	0.8
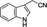	3‐indole acetonitrile	−	−	−	−	−	(+)	−	(+)	NIA	0.6
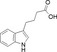	3‐indole butyric acid	−	−	−	−	−	−	(+)	−	0.8	NIA
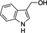	3‐indole methanol	−	−	−	(+)	+	+	+	+	0.6	0.4
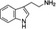	Tryptamine	−	−	−	−	−	−	−	−	NIA	NIA
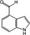	Indole‐4‐carbaldehyde	−	−	−	−	−	−	−	−	NIA	NIA
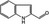	Indole‐2‐carbaldehyde	−	−	−	−	(+)	−	(+)	−	0.6	NIA
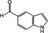	Indole‐5‐carbaldehyde	(+)	−	(+)	−	(+)	−	(+)	−	0.2	NIA
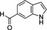	Indole‐6‐carbaldehyde	−	−	−	−	+	−	+	−	0.6	NIA
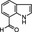	Indole‐7‐carbaldehyde	−	−	+	−	+	−	+	−	0.4	NIA
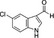	5‐chloroindole‐3‐carbaldehyde	−	(+)	+	+	+	+	+	+	0.4	0.2
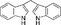	3,3′‐methylene indole	−	(+)	−	(+)	−	(+)	−	−	NIA	0.2
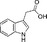	Indole acetic acid	−	−	−	−	−	−	−	−	NIA	NIA
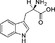	Tryptophan	−	−	−	−	−	−	−	−	NIA	NIA

Data were recorded 3 days after incubation. Symbol: +, inhibition; (+), partial inhibition; −, no inhibition; NIA, no inhibitory activity even at the highest concentration used in this study.

## Discussion

Antagonistic microbes usually produce inhibitory compounds or even enzymes to interfere with pathogen growth or signal transduction (Dong *et al*., 2004; Hayat *et al*., 2012). Our previous study has revealed that *Pseudomonas* sp. ST4 could produce active compounds to affect the hyphal growth and sexual mating of basidiospores of *S. scitamineum* (Liu *et al*., 2017). In this study, we purified two active compounds from the metabolites produced by *Pseudomonas* sp. ST4, which were identified respectively as 4‐hydroxybenzaldehyde and indole‐3‐carbaldehyde using NMR and mass spectrometry. The former inhibited the fungal hyphal growth at a concentration of 3 mM, and the latter interfered with the sexual mating at a concentration from 0.6 mM and the fungal growth at 2 mM (Table 2, Fig. 4). Pot trial against corn smut disease showed that indole‐3‐carbaldehyde could significantly reduce the disease incidence with about 94% control efficiency (Fig. 5, Table 1). This promising results of indole‐3‐carbaldehyde in antimating and disease control prompted us to screen for its derivatives with higher potency than the compound. Results led to identify indole as the most effective compound against the sexual mating of *S. scitamineum* with a MIC of 0.2 mM without any visible effect on the hyphal growth (Table 2). The findings suggest that indole group is critical for the antimating activity. In contrast, the position of carbaldehyde base on indole group appeared not so important. Any substitution at 3 or 4 or 6 position of indole would cause a substantial reduction in inhibitory activity (Table 2). The information would be useful for further design and optimization of inhibitors and for developing molecular probes to investigate the molecular mechanisms of inhibition.

Indole is widely found in nature, with numerous derivatives, in which, tryptophan is one of the essential amino acids, and IAA is a plant hormone. In addition, indole has also been widely used as a precursor or lead compound in the development of antibiotics or fungicides. Indole derivatives were shown to have potent antifungal or antibacterial activities. For example, 5‐nitro‐3‐formylindole exhibited a strong inhibitory activity on the growth of *Sclerotinia sclerotiorum* (Chen *et al*., 2016). Trichoderma produces indole‐3‐carboxaldehyde that induces camalexin (3‐thiazol‐2′‐yl‐indole) accumulation in Arabidopsis to resist the infection of *Botrytis cinerea* (Contreras‐Cornejo *et al*., 2011). In this study, we found that a soil bacterium *Pseudomonas* sp. ST4 also produced indole‐3‐carbaldehyde, which inhibited the sexual mating of *S. scitamineum* and *U. maydis*. Identification of indole and indole‐3‐carbaldehyde as antimating inhibitors in this study further expands the list of the biological functions and potential applications of indole derivatives.

Among the various structural derivatives of indole‐3‐carboxaldehyde tested in this study, three compounds, i.e., indole, 3‐methyl indole and indole‐5‐carboxaldehyde, showed antimating activity at a low concentration of 0.2 mM (Table 2). Indole and indole‐5‐carboxaldehyde did not show any cell toxicity against the fungal growth even at a concentration of 0.8 mM, whereas the compound 3‐methyl indole shared a similar antimating activity spectrum as indole except the cell toxicity at 0.8 mM. The properties of low cell toxicity and strong antimating potency of indole are highly valuable for developing environmental friendly anti‐infection agents. The molecular mechanism with which indole interferes with the fungal sexual mating especially the *b* locus expression remains to be determined. In this context, it is interesting to note that indole was found to inhibit bacterial quorum sensing (QS) signal transmission by interfering with the folding of a QS regulator (Kim and Park, 2013).

In summary, the findings from this study provide a molecular base with which *Pseudomonas* sp. ST4 interferes with the sexual mating of fungal pathogens *S. scitamineum* and *U. maydis. *In addition, identification of indole as a key functional group may aid our efforts for further investigation of molecular mechanisms by which fungal pathogens modulating the process of sexual mating, and for further design and developing novel anti‐infection reagents. Furthermore, it is interesting to note that *Pseudomonas* sp. ST4 produced multiple antimating and fungicide‐like molecules, which suggests that much more efforts are required for fully exploration and deep mining of potentially important bioactive molecules and valuable genetic resources from this interesting bacterial organism.

## Experimental procedures

### Strains and culture conditions

*Pseudomonas* sp. ST4 (deposited in the China Center for Type Culture Collection, CCTCC No: M2015526) was grown in Luria–Bertani (LB; per litre contains 5 g yeast extract, 10 g peptone and 10 g NaCl) broth or on potato dextrose agar (PDA, per litre contains 200 g potato, 20 g glucose and 18 g agar) plates for bioassay of bacterial antagonistic activity against fungal mating (Liu *et al*., 2017), unless otherwise indicated. *S. scitamineum* haploid cell lines MAT‐1 and MAT‐2, and *Ustilago maydis* haploid cell lines Umn9 and Umn10, were grown and maintained in YEPS medium (per litre contains 10 g yeast extract, 20 g peptone and 20 g sucrose) at 28°C or on solid medium containing 1.8% (wt/vol) agar (Yan *et al*., 2016).

### Thermal stability assessment of the *Pseudomonas* sp. ST4 active metabolites

Overnight bacterial culture of *Pseudomonas* sp. ST4 in LB broth was streaked in grid separated by 4 cm on PDA plates (13 × 13 cm) and incubated at 28°C for 48 h. Medium covered with ST4 cell lines was cut off and the remaining was collected and melted under 60°C, 20 ml of which was poured into a new petri dish (9 cm in diameter) for positive control. Another 20 ml was continuously melted under 100°C for 30 min and poured into another petri dish. After solidification, a mixture of *S. scitamineum* haploid cells MAT‐1 and MAT‐2 was spotted (0.5 μl of OD_600_ ≈ 1.5) on the plate. The mixture spotted on a fresh PDA plate was used as a negative control. All the plates were incubated at 28°C for 2 days until white hypha appeared on the negative control plate. The experiment was repeated in triplicates.

### Extraction of the active compounds from strain ST4

Overnight *Pseudomonas* sp. ST4 culture was spread on PDA plates (13 × 13 cm) and incubated at 28°C for 48 h, and bacterial cells were wiped off using sterilized gauze. Medium agar was chopped into small pieces and extracted 3 times with 3 volumes of EtOAc/MeOH/C_2_H_4_O_2_ (80: 15: 5) mixture for 2 h with constant shaking. Supernatants were collected and pooled together, and the organic solvents were removed using a rotary evaporator. Residues were then extracted 3 times with 3 volumes of MeOH, and the aqueous and organic phases were collected separately. The organic phase was evaporated to dryness under 45°C and weighted, a small amount of which was taken and dissolved in MeOH at a final concentration of 50 mg ml^−1^ for detection of antimating activity. The aqueous phase was then extracted 3 times with 3 volumes of C_4_H_10_O and the aqueous and C_4_H_10_O phases were collected separately. The C_4_H_10_O phase was evaporated to dryness and weighted, and dissolved in MeOH at a final concentration of 50 mg ml^−1^ for detection of antimating activity.

### Antimating activity bioassay

Haploid cell lines MAT‐1 and MAT‐2 were used for bioassay of the inhibitory activity of bacterial extracts or chemical compounds against the mating of the *S. scitamineum*. PDA plates were cut into separated slices (0.6 × 6 cm), and filter paper was cut into tablets of 6 × 6 mm in size and sterilized. A tablet was immersed into 100 μl of crude extract, and after drying, placed onto one end of the agar slice, and then, the mixture of cell lines MAT‐1 and MAT‐2 (0.5 μl of OD_600_ ≈ 1.5) was spotted on the slice at progressively further distances from the tablet. Tablet absorbed MeOH was assayed in the same way as a negative control. The plates were incubated at 28°C for 48 h until white hypha in the negative control grew to reach the edges of the slice. The bioassay was repeated for at least three times with triplicates.

### Separation and purification of the active compounds from *Pseudomonas* sp. ST4

Five grams of *Pseudomonas* sp. ST4 EtOAc extract was dissolved completely in suitable amount of MeOH, which were then mixed with 10 g of 60–100 mesh silica gels, stirred completely and air‐dried to remove extra MeOH. After that, these sample containing silica gels were added to a column, which was then connected to the Claricep Flash Silica (CS) standard silica gel column (Agela) in a Butch medium pressure filter. The sample was separated using gradient eluents of CHCl_3_:MeOH at a ratio of 100:0, 99:1, 98:2, 95:5, 90:10, 80:20 and 0:100 for 10 min at each gradient respectively (18 psi, 30 ml min^−1^ flow rate), to give seven elute fractions, which were combined into four fractions (Fr. ST4‐1 to Fr. ST4‐4) based on the pattern similarity in the thin layer chromatography (TLC) analysis (elution solvents, CHCl_3_:MeOH = 9:1; colour agent, 10% C_4_H_10_O_4_S) and the results of antimating bioassay. The collected fractions were evaporated to dryness and dissolved in MeOH at a final concentration of 50 mg ml^−1^ for bioassay of antimating activity as described above. Among the four fractions, Fr. ST4‐2 (732.8 mg) showed the strongest activity on inhibition of fungal mating. This fraction was thus further purified by semi‐preparative reversed‐phase HPLC by dissolving the residues in MeOH at a concentration of 100 mg ml^−1^. An aliquot of sample (100 μl) was injected, and eluted at a speed of 3 ml min^−1^ with a gradient solvent programme (CH_3_CN in H_2_O, 5–100%) for 30 min; then flushed with 100% CH_3_CN, followed by balancing the C18 semi‐preparative column (Phenomenex) for 5 min using 5% CH_3_CN. Elution peaks were monitored by a UV detector at OD_220_. Fractions were collected, vacuum dried, and dissolved in 5% MeOH for HPLC and TLC analysis, and for bioassay of antimating activity. The purified single peaks with antimating activity were vacuum dried and dissolved in MeOH or CD_3_OD for nuclear magnetic resonance (NMR) and mass spectrometry analyses.

### NMR and HRESIMS analyses

The ^1^H NMR and ^13^C NMR spectra were obtained at 600 MHz (Bruker Avance III 600). High‐resolution electrospray ionization mass spectroscopy (HRESIMS) analysis was performed with a Bruker maXis impact.

### Determination of antimating activity of purified compounds and synthetic chemicals

The pure compounds 4‐hydroxybenzaldehyde, indole‐3‐carbaldehyde and other derivatives were purchased from a commercial company (Shanghai Aladdin Biochemical Technology), and 100 mM of stock solutions was prepared in MeOH. Bioassay was conducted in a 24‐well plate with each well containing 1 ml PDA medium supplemented with a purified or purchased compound at various final concentrations as indicated. PDA medium supplemented with MeOH was used as a negative control. The mixture of MAT‐1 and MAT‐2 cells (0.5 μl at OD_600_ ≈ 1.5) was spotted onto the well after medium solidification. Plates were incubated at 28°C for 2 days until white hypha appeared on the negative control wells. The experiment was repeated for at least twice in triplicates.

### Pot trial of chemicals and *Pseudomonas* sp. ST4 on control of corn smut disease

Corn seeds were soaked in water for 24 h and grown in pots for 14 days till seedlings reaching about 15 cm in height. The fresh cultures of the haploid cell lines Umn9 and Umn10 of *U. maydis* and *Pseudomonas* sp. ST4 were prepared by inoculation of corresponding fungal or bacterial cell in YEPS medium, respectively, and grown overnight. The fungal and bacterial inocula were prepared by diluting the corresponding overnight cultures with the same medium to OD_600_ = 1.0 and OD_600_ = 0.5 respectively. Inoculation of corn seedlings was performed following the method described previously (Yan *et al*., 2016). Briefly, corn seedlings were inoculated with 500 μl of Umn9 and Umn10 cell mixture; Umn9 and Umn10 mixture with 4‐hydroxybenzaldehyde (3 mM, final concentration); Umn9 and Umn10 mixture with indole‐3‐carbaldehyde (3 mM, final concentration), *Pseudomonas* sp. ST4 culture and *Pseudomonas* sp. ST4 culture supplemented with 2% glucose respectively. The seedlings were kept outdoor for about 10 days before measurement of the disease incidence. The trial was conducted twice, and each treatment contains 20 seedlings of similar height.

## Conflict of interest

None declared.

## Supporting information


**Fig. S1.** Thermal stability of *Pseudomonas* sp. ST4 metabolites.
**Fig. S2.** Inhibitory effect of different crude extracts from strain ST4 on the sexual mating of MAT‐1 and MAT‐2.
**Fig. S3.** Bioassay with strain ST4 and its two active metabolic compounds on the sexual mating and hyphal growth of *U. maydis*.
**Fig. S4.** NMR analyses of fractions ST4‐2‐6 and ST4‐2‐8.

**Data S1**.Chromatogram of Fractions ST4‐1∼ST4‐3 with antifungal activity from C18 analytical column (Phenomenex) after the silica gel column separation.
