# Supplementary material for: Pseudomonas sp. ST4 produces variety of active compounds to interfere fungal sexual mating and hyphal growth
Source: Microb Biotechnol. 2018 Jun 21;13(1):107–17. doi: 10.1111/1751-7915.13289 (PMC6922531; doi:10.1111/1751-7915.13289)
Supplement: Supplementary file 1 — Fig. S1. Thermal stability of Pseudomonas sp. ST4 metabolites. Fig. S2. Inhibitory effect of different crude extracts from strain ST4 on the sexual mating of MAT‐1 and MAT‐2. Fig. S3. Bioassay with strain ST4 and its two active metabolic compounds on the sexual mating and hyphal growth of U. maydis. Fig. S4. NMR analyses of fractions ST4‐2‐6 and ST4‐2‐8. [file MBT2-13-107-s001.docx]

**Supporting information**

***Pseudomonas* sp. ST4 produces variety of active compounds to interfere fungal sexual mating and hyphal growth**

Shiyin Liu^1,2^, Fei He^1,2^, Nuoqiao Lin^1,2^, Yumei Chen^1,2^, Zhibin Liang^1,2^, Lisheng Liao^1,2^, Mingfa Lv^1,2^, Yufan Chen^1,2^, Shaohua Chen^1,2^, Jianuan Zhou^1,2^*, Lianhui Zhang^1,2^*,

*To whom correspondence should be addressed: [lhzhang01@scau.edu.cn;](mailto:lhzhang01@scau.edu.cn;)

[jianuanzhou@scau.edu.cn](mailto:jianuanzhou@scau.edu.cn;)

**
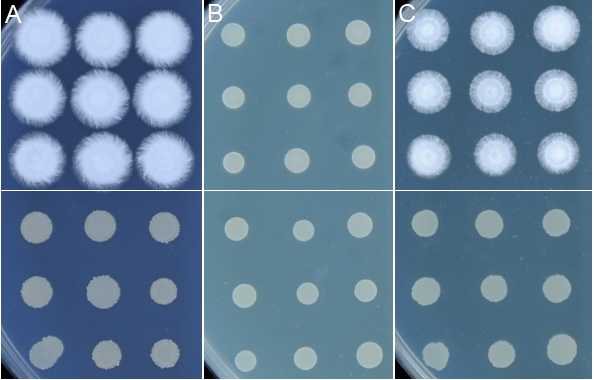
**

**Fig. S1** Thermal stability of *Pseudomonas* sp. ST4 metabolites. Mixture of MAT-1 and MAT-2 (top) and MAT-1 (bottom) on (A) PDA plate, (B) ST4 metabolite plate, and (C) ST4 metabolite plate after treatment at 100 ℃ for 30 min.


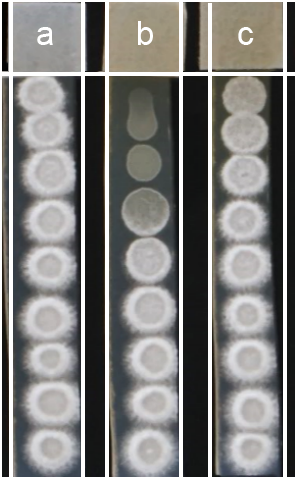


**Fig. S2** Inhibitory effect of different crude extracts from strain ST4 on the sexual mating of MAT-1 and MAT-2. The tablets were absorbed in 100 μL of MeOH as a negative control (a), EtOAc extract (b) and C_4_H_10_O extract (c) soluble in MeOH at a final concentration of 50 mg/mL, respectively.

**
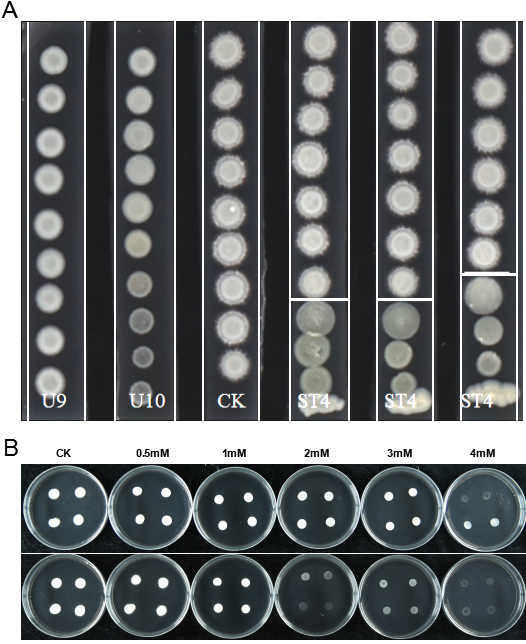
**

**Fig. S3** Bioassay with strain ST4, and its two active metabolic compounds on the sexual mating and hyphal growth of *U. maydis.* (A) Inhibitory effect of strain ST4 on the sexual mating of *U. maydis*. Haploid cells Umn9, Umn10, or their mixture (0.5 μL of OD_600_ ≈ 1.5) was spotted on PDA slices progressively. ST4 overnight culture was streaked on one end of the slices. LB medium was streaked as a negative control. The bioassay plate was incubated at 28 ^o^C for 2 d until white hypha appeared on the negative control. (B) Inhibitory effects of 4-hydroxybenzaldehyde (top) and indole-3-carboxaldehyde (bottom) on the sexual mating and hyphal growth of *U. maydis*. CK, PDA medium supplemented with MeOH. Results showed that 4-hydroxybenzaldehyde has no anti-mating activity but can influence the hyphal growth at the concentration of 4 mM and above; indole-3-carbaldehyde can interfere the fungal mating ranging from 2 mM to 4 mM.


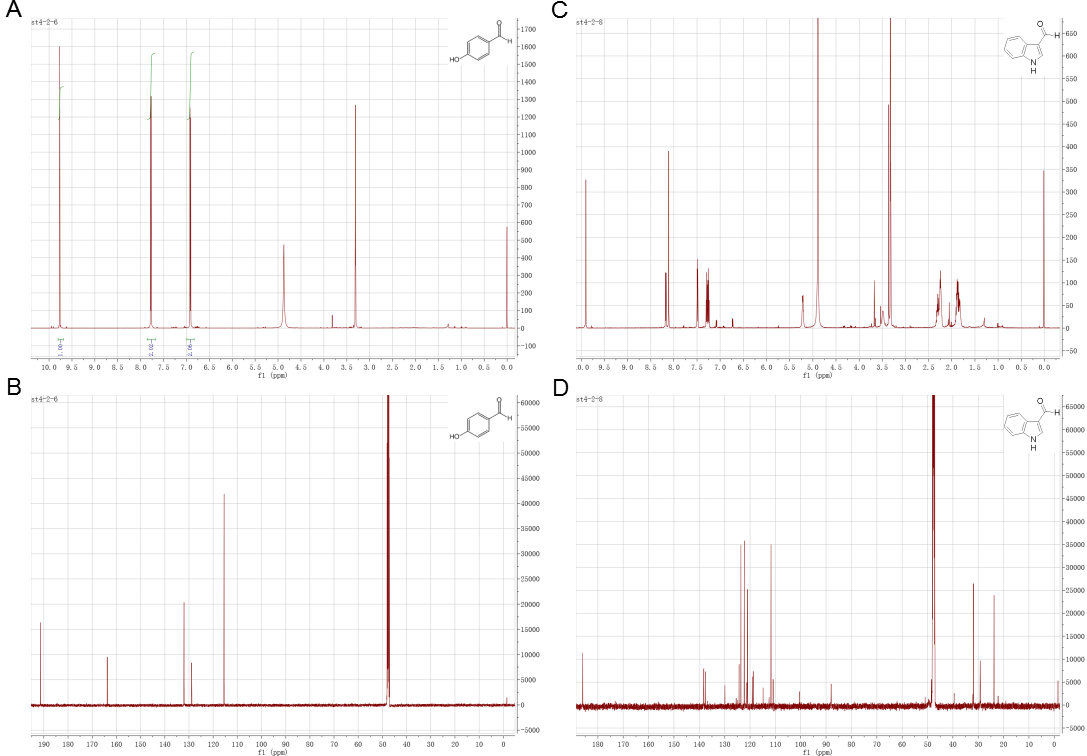


**Fig. S4** NMR analyses of fractions ST4-2-6 and ST4-2-8. (A) ^1^H-NMR map of Fr. ST4-2-6; (B) ^13^C-NMR map of Fr. ST4-2-6; (C) ^1^H-NMR map of Fr. ST4-2-8; (D) ^13^C-NMR map of Fr. ST4-2-8.
