# Supplementary material for: Pseudomonas sp. ST4 produces variety of active compounds to interfere fungal sexual mating and hyphal growth
Source: Microb Biotechnol. 2018 Jun 21;13(1):107–17. doi: 10.1111/1751-7915.13289 (PMC6922531; doi:10.1111/1751-7915.13289)
Supplement: Supplementary file 2 — Data S1.Chromatogram of Fractions ST4‐1∼ST4‐3 with antifungal activity from C18 analytical column (Phenomenex) after the silica gel column separation. [file MBT2-13-107-s002.pdf]

Data File C:\Chem32\3\Data\checkout\ST4-1 2014-12-15 11-02-25.D
Sample Name: ST4-1

===

Acq. Operator : SYSTEM
Sample Operator : SYSTEM
Acq. Instrument : LC1260+DAD   Location : 1
Injection Date : 12/15/2014 11:03:14 AM
Inj Volume : 1.000 µl
Acq. Method : C:\Chem32\3\Methods\lsy-ST4.M
Last changed : 12/15/2014 11:02:18 AM by SYSTEM
(modified after loading)
Analysis Method : C:\Chem32\3\Methods\5%-100%MeOH+H20 2017 04 07 .M
Last changed : 3/8/2018 10:48:09 AM by SYSTEM
(modified after loading)
Additional Info : Peak(s) manually integrated

DAD1 A, Sig=254,4 Ref=off (checkout\ST4-1 2014-12-15 11-02-25.D)

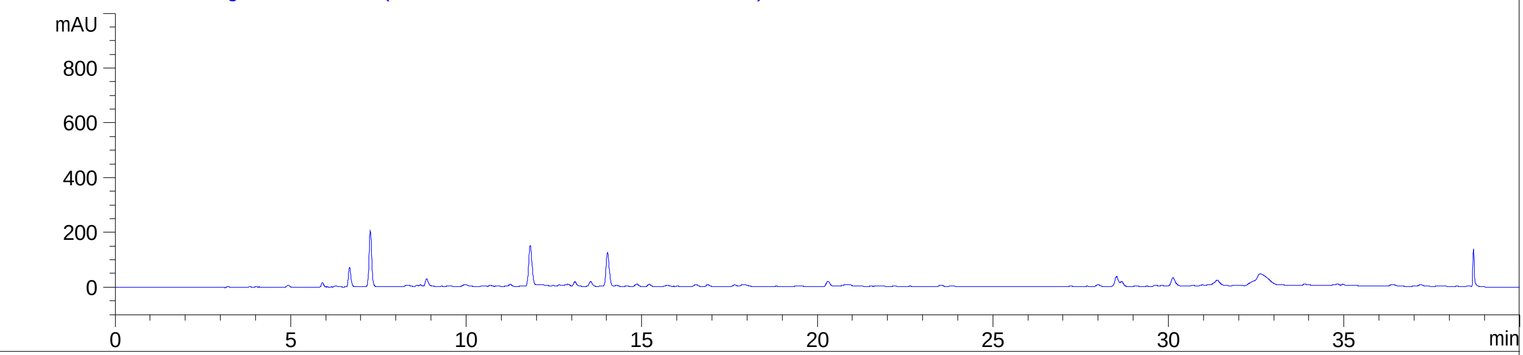

DAD1 B, Sig=202,4 Ref=off (checkout\ST4-1 2014-12-15 11-02-25.D)

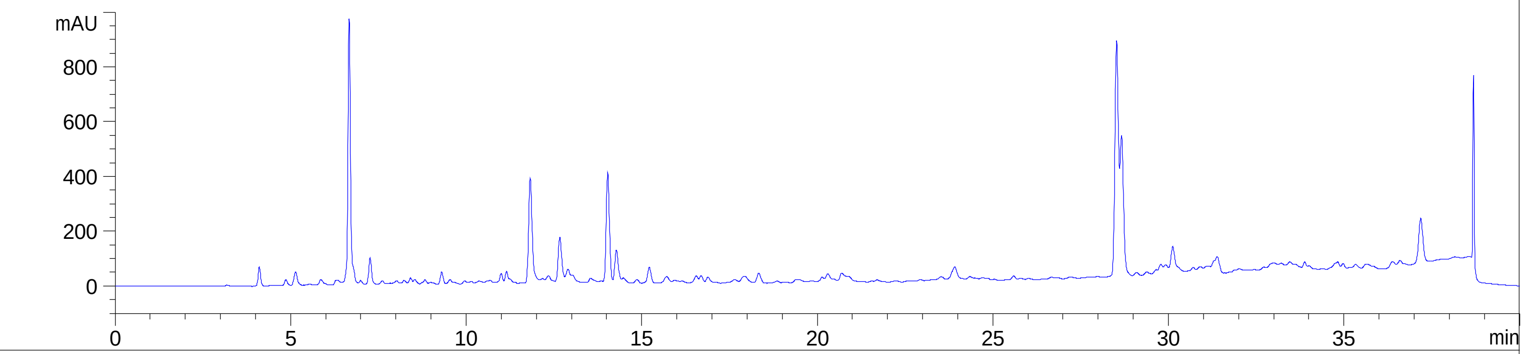

DAD1 C, Sig=210,4 Ref=off (checkout\ST4-1 2014-12-15 11-02-25.D)

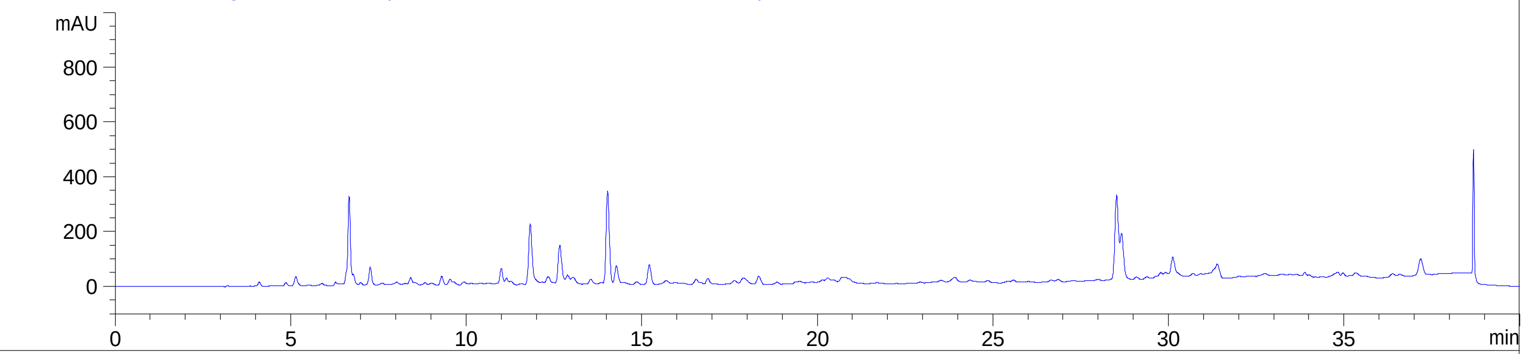

DAD1 D, Sig=230,4 Ref=off (checkout\ST4-1 2014-12-15 11-02-25.D)

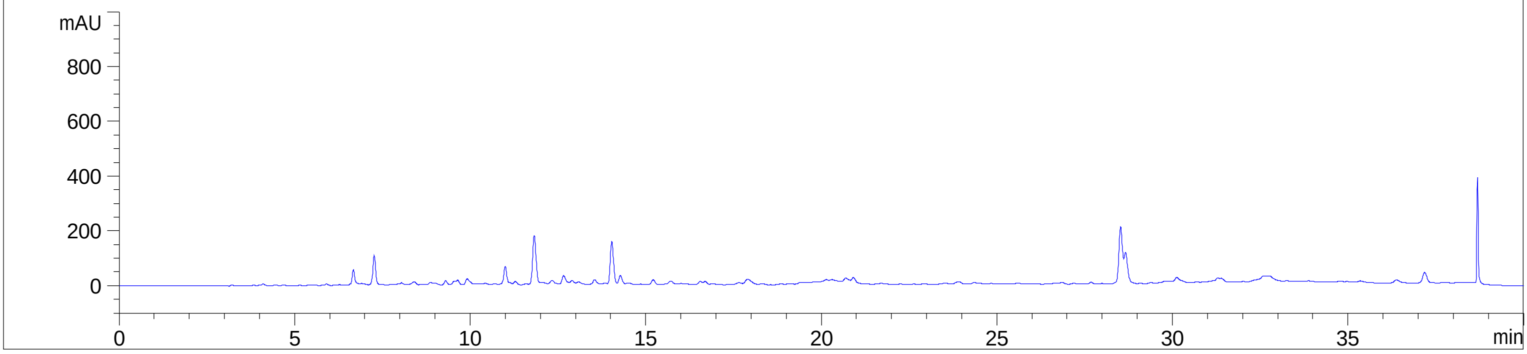

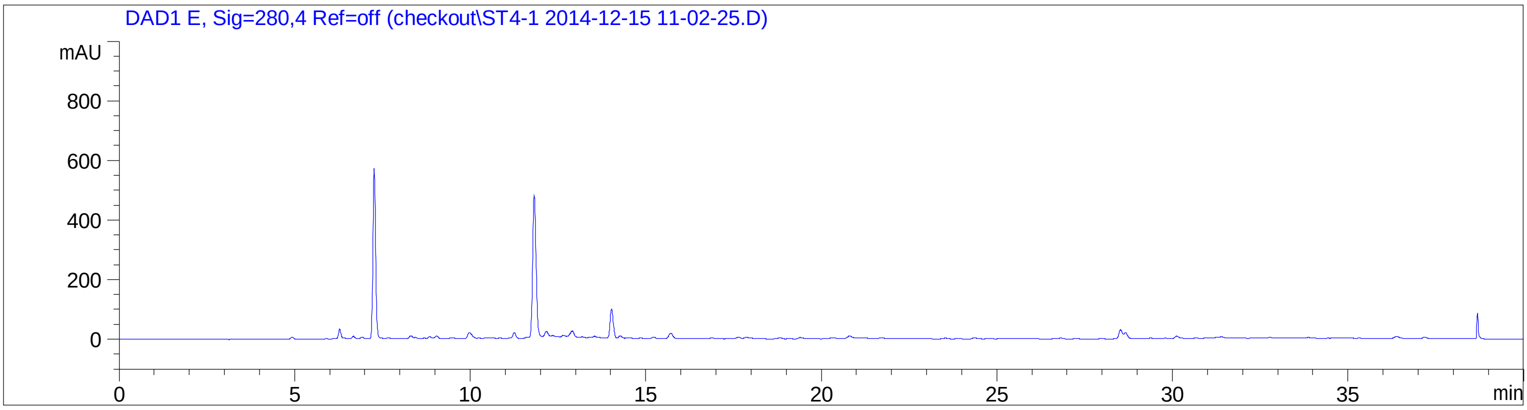

===

Area Percent Report with Performance

===

Multiplier : 1.0000
Dilution : 1.0000
Do not use Multiplier & Dilution Factor with ISTDs

Signal 1: DAD1 A, Sig=254,4 Ref=off

Signal 2: DAD1 B, Sig=202,4 Ref=off

Signal 3: DAD1 C, Sig=210,4 Ref=off

Signal 4: DAD1 D, Sig=230,4 Ref=off

Signal 5: DAD1 E, Sig=280,4 Ref=off

===

*** End of Report ***

Data File C:\Chem32\3\Data\checkout\ST4-2 2014-12-15 11-44-01.D
Sample Name: ST4-2

===

Acq. Operator : SYSTEM
Sample Operator : SYSTEM
Acq. Instrument : LC1260+DAD   Location : 2
Injection Date : 12/15/2014 11:44:47 AM
                Inj Volume : 1.000 µl
Acq. Method : C:\Chem32\3\Methods\lsy-ST4.M
Last changed : 12/15/2014 11:02:18 AM by SYSTEM
(modified after loading)
Analysis Method : C:\Chem32\3\Methods\5%-100%MeOH+H20   2017 04 07   .M
Last changed : 3/8/2018 10:47:16 AM by SYSTEM
(modified after loading)
Additional Info : Peak(s) manually integrated

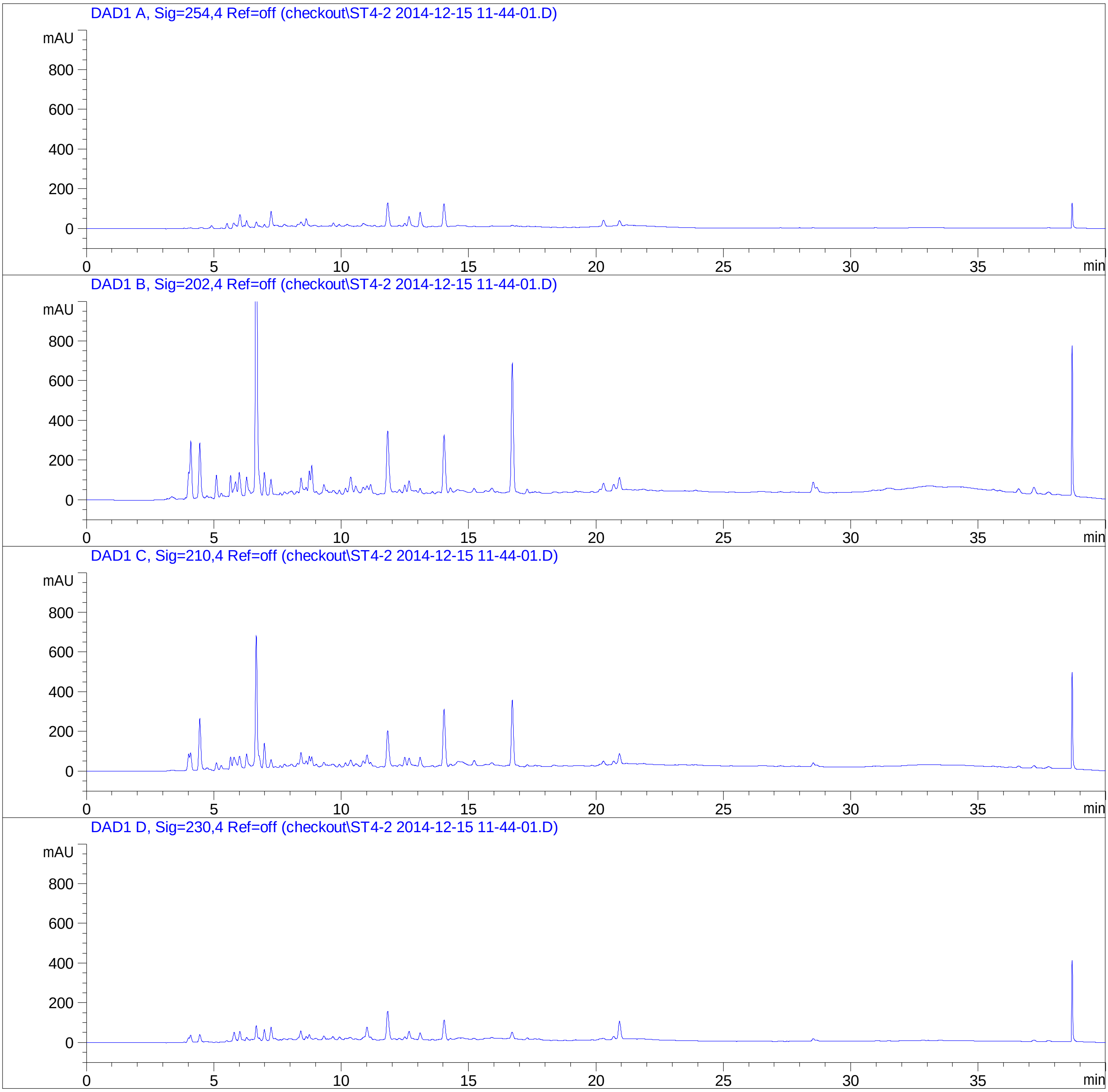

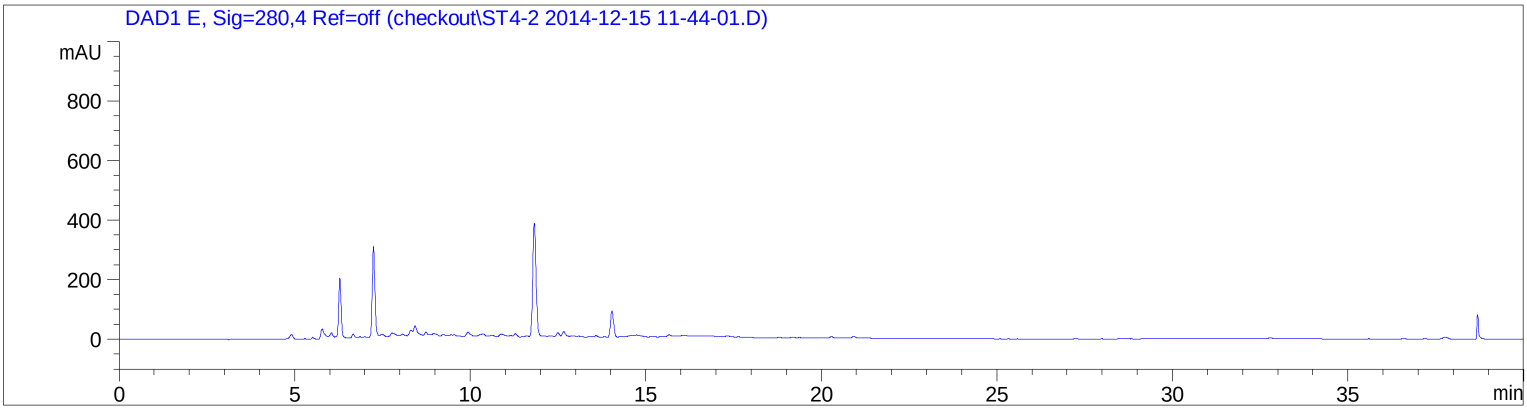

===

Area Percent Report with Performance

===

Multiplier : 1.0000
Dilution : 1.0000
Do not use Multiplier & Dilution Factor with ISTDs

Signal 1: DAD1 A, Sig=254,4 Ref=off

Signal 2: DAD1 B, Sig=202,4 Ref=off

Signal 3: DAD1 C, Sig=210,4 Ref=off

Signal 4: DAD1 D, Sig=230,4 Ref=off

Signal 5: DAD1 E, Sig=280,4 Ref=off

===

*** End of Report ***

Data File C:\Chem32\3\Data\checkout\ST4-3 2014-12-15 15-26-46.D
Sample Name: ST4-3

===

Acq. Operator : SYSTEM
Sample Operator : SYSTEM
Acq. Instrument : LC1260+DAD   Location : 3
Injection Date : 12/15/2014 3:27:35 PM
                                                Inj Volume : 5.000 µl
Acq. Method : C:\Chem32\3\Methods\lsy-ST4.M
Last changed : 12/15/2014 3:26:34 PM by SYSTEM
(modified after loading)
Analysis Method : C:\Chem32\3\Methods\5%-100%MeOH+H20   2017 04 07   .M
Last changed : 3/8/2018 10:44:39 AM by SYSTEM
(modified after loading)
Additional Info : Peak(s) manually integrated

DAD1 A, Sig=254,4 Ref=off (checkout\ST4-3 2014-12-15 15-26-46.D)

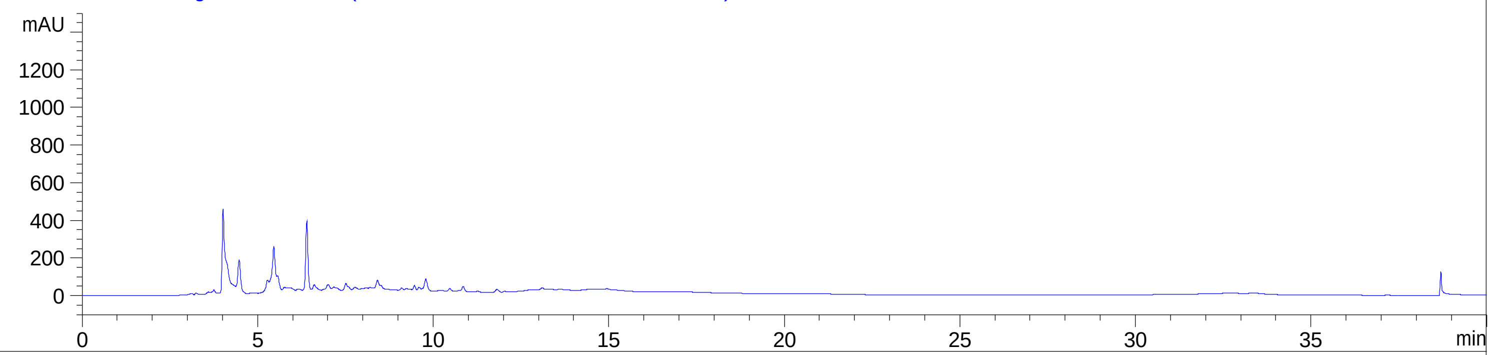

DAD1 B, Sig=202,4 Ref=off (checkout\ST4-3 2014-12-15 15-26-46.D)

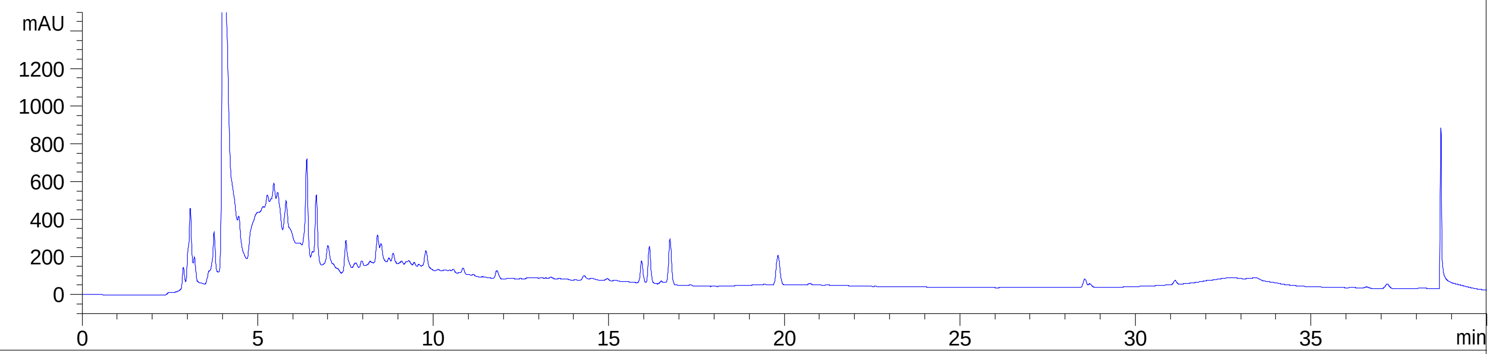

DAD1 C, Sig=210,4 Ref=off (checkout\ST4-3 2014-12-15 15-26-46.D)

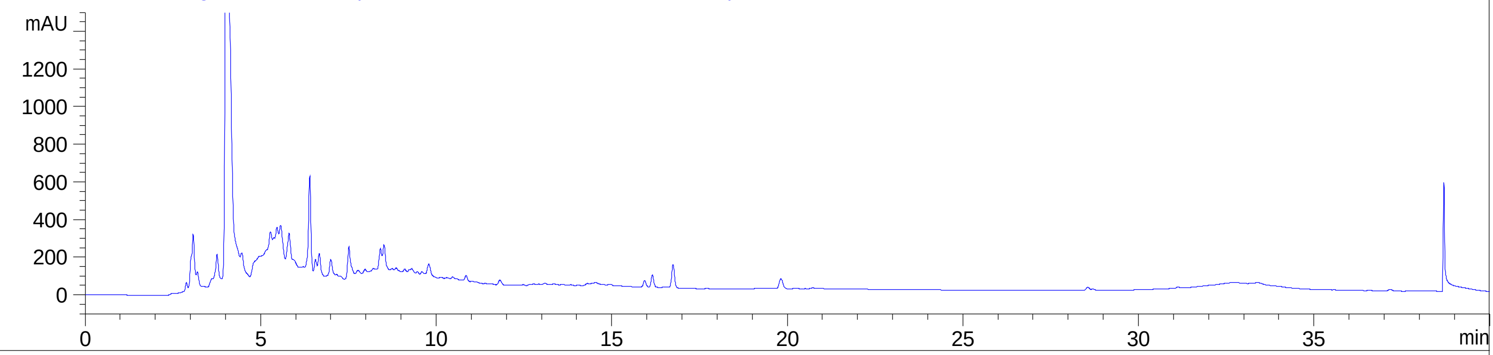

DAD1 D, Sig=230,4 Ref=off (checkout\ST4-3 2014-12-15 15-26-46.D)

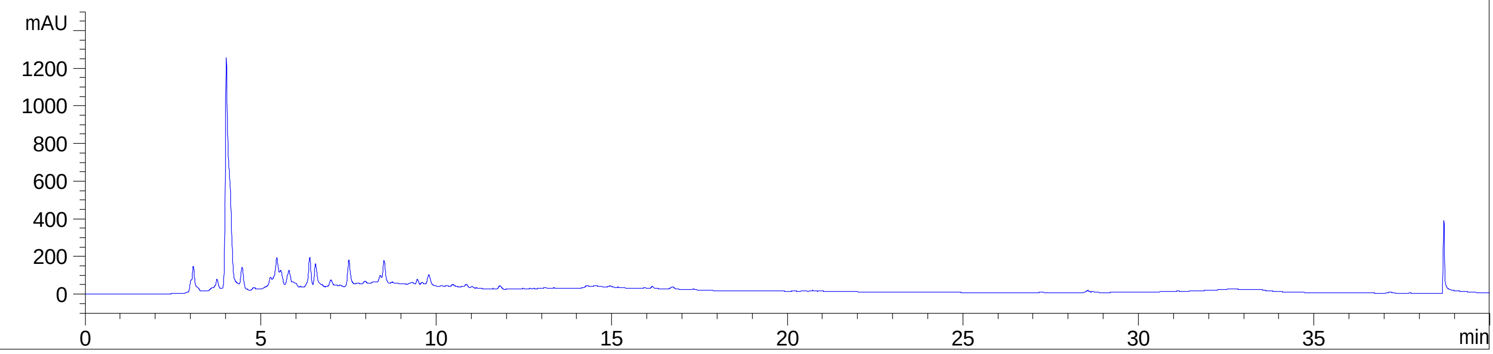

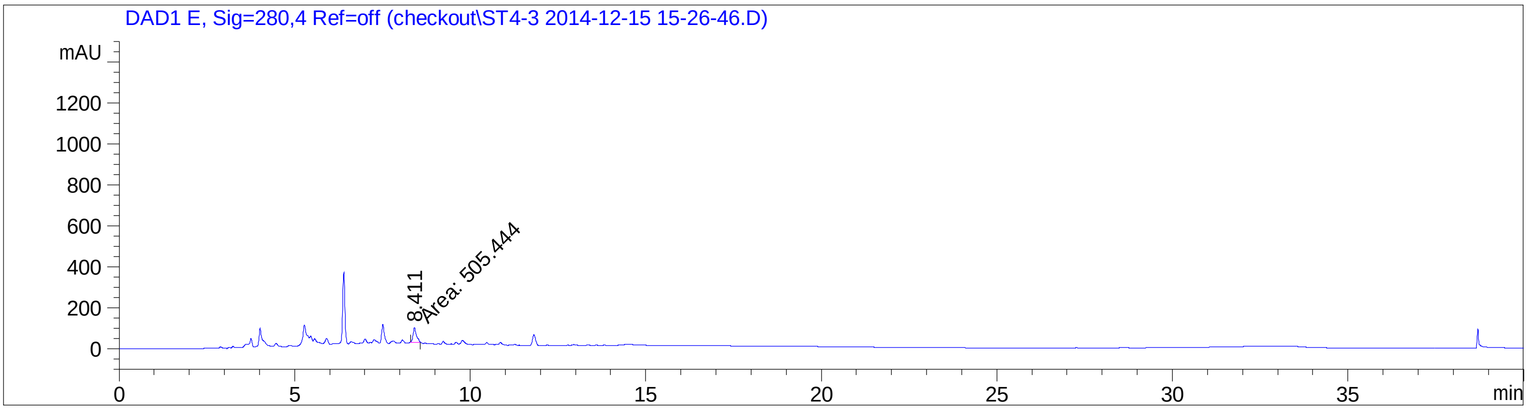

===

Area Percent Report

===

Sorted By : Signal
Multiplier : 1.0000
Dilution : 1.0000
Use Multiplier & Dilution Factor with ISTDs

Signal 1: DAD1 A, Sig=254,4 Ref=off

Signal 2: DAD1 B, Sig=202,4 Ref=off

Signal 3: DAD1 C, Sig=210,4 Ref=off

Signal 4: DAD1 D, Sig=230,4 Ref=off

Signal 5: DAD1 E, Sig=280,4 Ref=off

Peak #	RetTime [min]	Type	Width [min]	Area [mAU*s]	Height [mAU]	Area %
1	8.411	MM	0.1133	505.44449	74.34506	100.0000

Totals : 505.44449 74.34506

===

*** End of Report ***
